# mzML2ISA & nmrML2ISA: generating enriched ISA-Tab metadata files from metabolomics XML data

**DOI:** 10.1093/bioinformatics/btx169

**Published:** 2017-04-07

**Authors:** Martin Larralde, Thomas N Lawson, Ralf J M Weber, Pablo Moreno, Kenneth Haug, Philippe Rocca-Serra, Mark R Viant, Christoph Steinbeck, Reza M Salek

**Affiliations:** 1 École Normale Supérieure de Cachan, Cachan, France; 2 School of Biosciences, University of Birmingham, Birmingham, UK; 3 Phenome Centre Birmingham, University of Birmingham, Birmingham, UK; 4 European Molecular Biology Laboratory, European Bioinformatics Institute (EMBL-EBI), Wellcome Trust Genome Campus, Cambridge, UK; 5 University of Oxford e-Research Centre, Oxford, UK; 6 Institute for Inorganic and Analytical Chemistry, Friedrich-Schiller-University, Lessingstr. 8, Jena, Germany

## Abstract

**Summary:**

Submission to the MetaboLights repository for metabolomics data currently places the burden of reporting instrument and acquisition parameters in ISA-Tab format on users, who have to do it manually, a process that is time consuming and prone to user input error. Since the large majority of these parameters are embedded in instrument raw data files, an opportunity exists to capture this metadata more accurately. Here we report a set of Python packages that can automatically generate ISA-Tab metadata file stubs from raw XML metabolomics data files. The parsing packages are separated into mzML2ISA (encompassing mzML and imzML formats) and nmrML2ISA (nmrML format only). Overall, the use of mzML2ISA & nmrML2ISA reduces the time needed to capture metadata substantially (capturing 90% of metadata on assay and sample levels), is much less prone to user input errors, improves compliance with minimum information reporting guidelines and facilitates more finely grained data exploration and querying of datasets.

**Availability and Implementation:**

mzML2ISA & nmrML2ISA are available under version 3 of the GNU General Public Licence at https://github.com/ISA-tools. Documentation is available from http://2isa.readthedocs.io/en/latest/.

**Supplementary information:**

Supplementary data are available at *Bioinformatics* online.

## 1 Introduction

MetaboLights, a database of experimental and derived metabolomics data (Kale *et al.*, 2016), currently requires studies to be submitted in the ISA-Tab file format (Sansone *et al.*, 2008), a hierarchical structure consisting of three components (Investigation, Study and Assay). ISA-Tab allows experimentalists to describe and record metadata in a simple format that aids reproducibility and shareability of experimental data and results. In addition to being a requirement for MetaboLights, the format is used for data centric journals (*GigaScience, Scientific Data*) and can facilitate data analysis (González-Beltrán *et al.*, 2014; Lekschas and Gehlenborg, 2016).

Data acquisition within a metabolomics experiment involves a wide range of instrument and data pre-processing parameters. Monitoring and recording those parameters is essential to maximize the reproducibility of experimental data. Currently MetaboLights utilizes the ISA software suite (Rocca-Serra *et al.*, 2010), particularly ISAcreator, to capture this information in ISA-Tab syntactic elements. Manual entry of >40 potential parameters and associated ontology references for MetaboLights submission can be a laborious, time-consuming and error-prone process. However, the majority of this instrument metadata exists already within the instrument vendor data files and corresponding open source data formats.

The use of freely available open source data formats for both mass spectrometry (MS) and nuclear magnetic resonance (NMR) spectroscopy is advantageous for software development as it allows a single route to access instrument data and allows metadata to be standardized between vendors. The open source MS file format mzML (Martens *et al.*, 2011) was created in 2008 as part of the Human Proteome Organization (HUPO) Proteomics Standards Initiative working group for mass spectrometry (PSI-MS). The mzML format amalgamated many of the benefits of the older MS formats (mzData (Orchard *et al.*, 2004)) and mzXML (Pedrioli *et al.*, 2004) while adding new features. Importantly, the mzML format has a MS controlled vocabulary and is easily extendable (Martens *et al.*, 2011). The imzML format is a relatively recent extension designed specifically for imaging MS (Schramm *et al.*, 2012). The nmrML file format is a newly developed file format for NMR data, while not an extension of mzML, it is built around the same principles (http://nmrml.org/).

Through the use of python packages, we automated both the extraction of the instrument metadata and the subsequent population of an ISA-Tab structure. In so doing, we aim to both remove the submission bottleneck and help ensure the experimental data is reusable, sharable and standards compliant (Salek *et al.*, 2015).

## 2 Materials and methods

### 2.1 ISA-Tab

The ISA-Tab structure consists of three file types. The Investigation file describes the overall objectives of a project as well as defining factors, protocols and parameters. The Study files describe the subjects studied and their characteristics and sampling methods. For every Study file there are one or more associated Assay files describing a measurement (e.g. metabolic profiling) and the technique (e.g. NMR). The instrument metadata extracted from either mzML, imzML or nmrML files are predominately used to automatically populate fields within the Assay file(s).

### 2.2 Implementation and availability

The mzML2ISA & nmrML2ISA software can be used as an application programming interface (API), via the command line interface (CLI) or via a graphical user interface (GUI). To allow integration into Galaxy, a popular workflow platform for metabolomics and other ’omic data analysis (Afgan *et al.*, 2016), the python packages have been wrapped into a Galaxy compatible tool format. Additionally, mzML2ISA & nmrML2ISA are available as tools within the PhenoMeNal Galaxy public instance and as Docker Containers as detailed in the supplementary materials. See Supplementary Table S1 for full list of nmrML2ISA & mzML2ISA software and Supplementary section S2 for further implementation details. Instruction on how to use the tools and examples for common use cases can be found in the documentation http://2isa.readthedocs.io/en/latest/.

### 2.3 Workflow

**Fig. 1:**
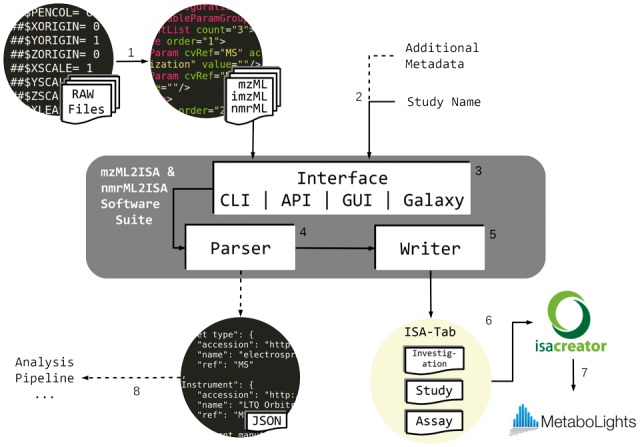
Schematic diagram and workflow of mzML2ISA & nmrML2ISA software suite. 1) Experimental vendor raw files are converted into an open source XML equivalent. 2) A user provides experimental metadata (minimally a study identifier). 3) The additional metadata and open source XML raw files are submitted to the mzML2ISA & nmrML2ISA software through either a CLI, API, GUI or Galaxy interface. The time to complete this step is dependent on the extent of metadata provided. 4) Metadata is extracted from XML files. 5) ISA-Tab structure is generated with a large number of fields automatically populated. Steps 4 and 5 take approximately 45 seconds for 50 XML files. 6) The remaining fields are then populated manually using the standard ISAcreator software. 7) The completed ISA-Tab structure can then be submitted to MetaboLights. 8) Additionally, the parsing components of mzML2ISA & nmrML2ISA can be used as standalone Python packages to extract metadata as either a python dictionary or JSON for integration in other analysis pipelines

Due to the nature of the analytical technologies and structural differences between MS and NMR file formats, the software described here is divided into mzML2ISA (encompassing mzML and imzML formats) and nmrML2ISA (nmrML format only). However, the same workflow applies for both technologies (see Fig. 1).

From a user supplied collection of XML based data files and some experimental information (minimally a study identifier), the metadata are extracted from the XML data files. The Python Pronto package (https://pypi.python.org/pypi/pronto) is used to extract parameters referring to their accession number within either the HUPO PSI-MS ontology (Mayer *et al.*, 2013), imagingMS ontology (Schramm *et al.*, 2012) or nmrCV (http://nmrml.org/).

Some instrument metadata is not available within the XML file format (e.g. chromatography parameters); such meta data can be added by the user manually. Additionally, study design or wet lab experimental metadata (e.g. sample preparation details) can also be added by the user manually. The GUIs developed provide the easiest route for users who wish to provide additional metadata at this stage.

The metadata is then converted into the ISA-Tab file format where a large number of fields are automatically populated. Depending on the level of additional metadata already provided by the user, the ISA-Tab files may require review and expansion to meet annotation requirements (Fiehn *et al.*, 2007; Sansone *et al.*, 2007). These remaining fields can be manually added through the ISAcreator tool.

The metadata generated from the XML file parsing is stored as a Python dictionary that can be rendered in JSON (JavaScript Object Notation), making it accessible to other software tools and analysis independent of ISA-Tab generation.

See Supplementary section S2 for full details of all MetaboLights studies used for assessing software in this paper.

## 3 Results

### 3.1 Extracted experimental metadata

Up to 23, 42 and 46 instrument metadata terms for mzML, imzML and nmrML respectively are automatically extracted and parsed into the assay component of the ISA-Tab structure.

Extracted terms for the XML files include generic instrument descriptors (e.g. the instrument name, manufacturer and software), data transformation descriptors (e.g. file conversion details) and platform specific descriptors (e.g. mass analyzer, detector and m/z range for MS derived XML files). Where possible, extracted terms from each file format should be found within a relevant controlled vocabulary. Full details of the extracted terms can be found at http://2isa.readthedocs.io/en/latest/ (see the extracted terms sections).

### 3.2 MetaboLights case studies

The mzML2ISA & nmrML2ISA packages have parsed and created ISA-Tab structures for all MetaboLights studies that have associated mzML, imzML, nmrML files. The resulting ISA-Tab structures have then been successfully validated using the ISAvalidator (https://github.com/ISA-tools/ISAvalidator-ISAconverter-BIImanager) tool. See Supplementary Table S2 for details of the 21 studies tested.

Using sample sets of 50 XML files (either mzML, imzML or nmrML) derived from MetaboLights, XML to ISA-Tab conversion is completed in less than 45 seconds. The exact time will vary depending on file type and size. See Supplementary Table S3 for details.

## 4 Discussion

The mzML, imzML and nmrML data file formats used in metabolomics provide a parameter rich, technical layer of metadata that we have exploited here to both improve the reliability of MetaboLights submissions and increase the ease and speed of submission. Additionally, the generated ISA-Tab structures can be used with subsequent downstream analysis with software such as Risa (González-Beltrán *et al.*, 2014) and the Refinery Platform (Lekschas and Gehlenborg, 2016).

However, to reach core information for metabolomics reporting compliance (CIMR) (BioSharing: Bsg-s000175), derived from the Metabolomics Standards Initiative (Fiehn *et al.*, 2007; Sansone *et al.*, 2007), a range of distinct descriptors still have to be reported into the ISA-Tab structure. Typically, these are experimental design key descriptors (predictor variables, replicate information, study group information), subject to sample relationships, as well as biological sample characteristics, and sample separation procedures if relevant (e.g chromatography). The development of the ISA-API (https://github.com/ISA-tools/isa-api) will further facilitate the automation of these missing descriptors, especially regarding experimental design, and aid in reducing the barrier and time required to submit metabolomics data to repositories. Indeed, instrument vendor raw files may harbour many more parameters that are often absent in the open source equivalent files.

Metadata extraction and integration into an open source file format can however be an involved process to implement for each instrument vendor API. Additionally, licensing issues can limit full exploitation of the vendor raw files. Further work that brings together the metabolomics and proteomics community, along with the instrument vendors, could help identify and integrate useful but missing parameters into the appropriate open source format.

## Supplementary Material

Supplementary Data
