# Supplementary material for: mzML2ISA & nmrML2ISA: generating enriched ISA-Tab metadata files from metabolomics XML data
Source: Bioinformatics. 2017 Apr 7;33(16):2598–600. doi: 10.1093/bioinformatics/btx169 (PMC5870861; doi:10.1093/bioinformatics/btx169)
Supplement: Supplementary Data [file supplemental_09022017_btx169.pdf]

SUPPLEMENTAL DATA
mzML2ISA & nmrML2ISA: Generating enriched ISA-Tab metadata files from metabolomics XML data

Martin Larralde[1†], Thomas N. Lawson[2†], Ralf J. M. Weber[2,3], Kenneth Haug[4], Philippe Rocca-Serra[5], Pablo Moreno[4], Mark R. Viant[2,3], Christoph Steinbeck[4], and Reza M. Salek[4]

[1]École Normale Supérieure de Cachan, 61 Avenue du Président Wilson, 94230 Cachan, France

[2]School of Biosciences, University of Birmingham, Birmingham, B15 2TT, UK

[3]Phenome Centre Birmingham, University of Birmingham, Birmingham, B15 2TT, UK

[4]European Molecular Biology Laboratory, European Bioinformatics Institute (EMBL-EBI), Wellcome Trust Genome Campus, Cambridge, UK

[5]University of Oxford e-Research Centre, 7 Keble Road, OX1 3QG, Oxford, UK

[†]Authors contributed equally

February 9, 2017

Contents

1 Software Summary **2**

2 mzML2ISA & nmrML2ISA Implementation **3**

3 MetaboLights Study Details **4**

4 Benchmarking Results **6**

1 Software Summary

Table 1: Software suite

Interface	Technology	Software Name	XML Format
CLI and API	MS	mzML2ISA	mzML, imzML
	NMR	nmrML2ISA	nmrML
GUI	MS	mzML2ISA-qt	mzML
	MS	imzML2ISA-qt	imzML
	NMR	nmrML2ISA-qt	nmrML
Galaxy tool	MS	mzML2ISA-galaxy	mzML, imzML
	NMR	nmrML2ISA-galaxy	nmrML

API: application program interface; CLI: command line interface; GUI: graphical user interface; MS: mass spectrometry; NMR: nuclear magnetic resonance

2 mzML2ISA & nmrML2ISA Implementation

All vendor raw data files require conversion into their respective open source file format: mzML (Martens *et al.*, 2011) for mass spectrometry (MS), imzML (Schramm *et al.*, 2012) for imaging MS and nmrML (http://nmrml.org/) for nuclear magnetic resonance (NMR). Converters are freely available to convert instrument vendor raw files to open source equivalents, see (http://nmrml.org/converter/) for NMR files and MSconvert (Chambers *et al.*, 2012) for MS files.

Both mzML2ISA & nmrML2isa packages are fully compatible with Python 2.7 and 3.5 (CPython implementation). Continuous Integration relies on Travis CI (https://travis-ci.org/) and AppVeyor (https://appveyor.com/) for automated building and testing of packages on multiple operating systems, using MetaboLights data repository as model data (available open and free via FTP). Development is being carried out on the experimental branch of the Github repository of each project while distribution is assured via Github releases and PyPI (https://pypi.python.org/pypi). Code reference for citation is available via Zenodo DOIs (https://zenodo.org/). Documentation is generated using Sphinx (http://www.sphinx-doc.org/) and hosted on readthedocs.io (https://readthedocs.org/). The mzML2ISA, nmrML2ISA and imzML2ISA GUIs are built using PyQt5 (https://pypi.python.org/pypi/PyQt5) , and only work with Python 3.

The Pronto python package (https://pypi.python.org/pypi/pronto), capable of parsing both OwlXML (Dean *et al.*, 2004; Hitzler *et al.*, 2009) and OBO ontology (Hancock *et al.*, 2004; Smith *et al.*, 2007) file formats, was developed to facilitate the extraction of the relevant ontologies from each format. Pronto provides a unique API for both formats, allowing to browse ontology terms and easily get access to a terms parents or children. Pronto enables mzML2ISA & nmrML2ISA to extract parameters referring to their accession number within either the PSI-MS ontology (Mayer *et al.*, 2013) or the imagingMS ontology (Schramm *et al.*, 2012), but also alerts to possible errors within the mzML files concerning the way the controlled vocabulary terms were written. The metadata information stored as a Python dictionary that can be rendered in JSON (JavaScript Object Notation), making it accessible to many software tools.

Packages are available as independent docker containers through the PhenoMeNal project, see below for the docker command line calls:

```
$ docker pull container-registry.phenomenal-h2020.eu/phnmnl/mzml2isa
$ docker pull container-registry.phenomenal-h2020.eu/phnmnl/nmrml2isa
```

Tools are available as well as part of the PhenoMeNal Galaxy Virtual Research Environment public instance at http://public.phenomenal-h2020.eu/.

3 MetaboLights Study Details

Table 2: MetaboLight Studies with XML Open Source Raw File Formats

Study ID	Title	XML
MTBLS1	A metabolomic study of urinary changes in type 2 diabetes in human compared to the control group	nmrML
MTBLS32	Lipid mediators of inflammation in BALF 6-19 days after infection with influenza.	mzML
MTBLS36	Metabolic differences in ripening of Solanum lycopersicum 'Ailsa Craig' and three monogenic mutants	mzML
MTBLS38	Metabolite Standards for the development and validation of MassCascade	mzML
MTBLS67	Metabolomic Analysis of Fission Yeast at the Onset of Nitrogen Starvation	mzML
MTBLS87	Unexpected similarities between the Schizosaccharomyces and human blood metabolomes, and novel human metabolites (Blood fraction)	mzML
MTBLS88	Unexpected similarities between the Schizosaccharomyces and human blood metabolomes, and novel human metabolites (Blood plasma and RBC fractions)	mzML
MTBLS125	Distribution of RESV and its metabolite peaks in mouse tissues after oral and skin administration	mzML
MTBLS126	Absorption efficiency of RESV through mouse skin using 3 bases in different tissues	mzML
MTBLS127	Resveratrol metabolism in HepG2 (human hepatocytes), HaCaT (human keratinocytes), and C2C12 (mouse myoblasts)	mzML
MTBLS137	MetaDB a Data Processing Workflow in Untargeted MS-Based Metabolomics Experiments	mzML
MTBLS140	Metabolome analysis via an HPLC-ESI-MS-based experimental and computational pipeline for chronic nephron toxicity profiling	mzML
MTBLS228	†Untargeted extraction of metabolites 13C labeling profiles from time course labeling switch experiment	mzML
MTBLS229	†Untargeted extraction of metabolites 13C labeling profiles from time course labeling switch experiment	mzML
MTBLS263	Individual variability in human blood metabolites identifies age-related differences - determination of coefficients of variation for each metabolite (3 injections of same sample, 3 independent sample preparations).	mzML
MTBLS265	Individual variability in human blood metabolites identifies age-related differences (30 persons, whole blood data).	mzML

Table 2: MetaboLight Studies with XML Open Source RAW File Formats (continued)

Study ID	Title	XML
MTBLS266	Individual variability in human blood metabolites identifies age-related differences (30 persons, plasma data).	mzML
MTBLS273	Metabolic phenotyping of ex-vivo breast samples by DESI mass spectrometry imaging	imzML
MTBLS289	Analysis of colorectal adenocarcinoma tissue samples by DESI mass spectrometry imaging	imzML
MTBLS315	Towards improving point-of-care diagnosis of non-malaria febrile illness: a metabolomics approach	mzML
MTBLS341	Piriformospora indica stimulates root metabolism of Arabidopsis thaliana	mzML

† Titles are the same but the studies and associated files are unique

4 Benchmarking Results

Assessment ran on Ubuntu 16.04.1 LTS 64bit Virtual Machine (Virtual Box 5.0.3) with a maximum of 2 CPUs and 8 GB of memory. The times shown are a mean calculated from 5 repeat measurements. mzML2ISA v(0.5.0) was used for .mzML and .imzML files, nmrML2ISA v(0.3.0) was used for nmrML files.

Table 3: Benchmarking of Sample XML Data Files

MetaboLights ID	Mean File size (MB)	Python version	CPUs	Mean time (Seconds)	StDev
MTBLS1 (nmrML)	0.51	2.7	1	0.82	0.15
			2	0.77	0.03
		3.5	1	0.70	0.03
			2	0.70	0.02
MTBLS127 (mzML)	78.8	2.7	1	26.3	0.89
			2	18.2	0.30
		3.5	1	25.6	0.40
			2	18.6	0.25
MTBLS137 (mzML)	51.9	2.7	1	28.4	2.03
			2	20.5	0.34
		3.5	1	26.1	0.32
			2	20.5	0.44
MTBLS273 (imzML)	7.8	2.7	1	16.2	0.27
			2	15.8	0.53
		3.5	1	16.2	0.19
			2	14.5	0.13
MTBLS289 (imzML)†	21.4	2.7	1	42.5	0.68
			2	44.6	0.91
		3.5	1	43.5	1.25
			2	39.5	1.08
MTBLS315 (mzML)	25.6	2.7	1	16.7	0.43
			2	13.4	0.41
		3.5	1	16.1	0.31
			2	14.3	0.59

† Profile and centroid .imzML pairs of files were available for each technical run. The assessment here used 25 .imzML (profile) and 25 .imzML (centroid) file pairs, i.e. a total of 50 .imzML files. The files were merged together to form a single entry (row) for each pair in the ISA assay file.

References

Chambers, M. C., Maclean, B., Burke, R., Amodei, D., Ruderman, D. L., Neumann, S., Gatto, L., Fischer, B., Pratt, B., Egertson, J., Hoff, K., Kessner, D., Tasman, N., Shulman, N., Frewen, B., Baker, T. A., Brusniak, M.-Y., Paulse, C., Creasy, D., Flashner, L., Kani, K., Moulding, C., Seymour, S. L., Nuwaysir, L. M., Lefebvre, B., Kuhlmann, F., Roark, J., Rainer, P., Detlev, S., Hemenway, T., Huhmer, A., Langridge, J., Connolly, B., Chadick, T., Holly, K., Eckels, J., Deutsch, E. W., Moritz, R. L., Katz, J. E., Agus, D. B., MacCoss, M., Tabb, D. L., and Mallick, P. (2012). A cross-platform toolkit for mass spectrometry and proteomics. *Nat. Biotechnol.*, **30**(10), 918–920.

Dean, M., Schreiber, G., Bechhofer, S., van Harmelen, F., and others (2004). OWL web ontology language reference. *W3C Recommendation*.

Hancock, J. M., Hancock, J. M., and Zvelebil, M. J. (2004). OBO foundry. In *Dictionary of Bioinformatics and Computational Biology*. John Wiley & Sons, Ltd.

Hitzler, P., Krötzsch, M., Parsia, B., Patel-Schneider, P. F., and others (2009). OWL 2 web ontology language primer. *W3C*.

Martens, L., Chambers, M., Sturm, M., Kessner, D., Levander, F., Shofstahl, J., Tang, W. H., Römpp, A., Neumann, S., Pizarro, A. D., and Others (2011). mzML—a community standard for mass spectrometry data. *Mol. Cell. Proteomics*, **10**(1), R110–000133.

Mayer, G., Montecchi-Palazzi, L., Ovelleiro, D., Jones, A. R., Binz, P.-A., Deutsch, E. W., Chambers, M., Kallhardt, M., Levander, F., Shofstahl, J., Orchard, S., Vizcaíno, J. A., Hermjakob, H., Stephan, C., Meyer, H. E., Eisenacher, M., and HUPO-PSI Group (2013). The HUPO proteomics standards initiative- mass spectrometry controlled vocabulary. *Database*, **2013**, bat009.

Schramm, T., Hester, A., Klinkert, I., Both, J.-P., Heeren, R. M. A., Brunelle, A., Laprévote, O., Desbenoit, N., Robbe, M.-F., Stoeckli, M., Spengler, B., and Römpp, A. (2012). imzML — a common data format for the flexible exchange and processing of mass spectrometry imaging data. *J. Proteomics*, **75**(16), 5106–5110.

Smith, B., Ashburner, M., Rosse, C., Bard, J., Bug, W., Ceusters, W., Goldberg, L. J., Eilbeck, K., Ireland, A., Mungall, C. J., OBI Consortium, Leontis, N., Rocca-Serra, P., Ruttenberg, A., Sansone, S.-A., Scheuermann, R. H., Shah, N., Whetzel, P. L., and Lewis, S. (2007). The OBO foundry: coordinated evolution of ontologies to support biomedical data integration. *Nat. Biotechnol.*, **25**(11), 1251–1255.
